# Randomized controlled trial of reinsemination strategies in dairy cows diagnosed nonpregnant using color flow Doppler ultrasonography on d 21 after insemination

**DOI:** 10.3168/jdsc.2021-0149

**Published:** 2021-10-09

**Authors:** J. Dubuc, V. Fauteux, J.-P. Roy, J. Denis-Robichaud, M. Rousseau, S. Buczinski

**Affiliations:** Faculté de médecine vétérinaire, Université de Montréal, 3200, rue Sicotte, Saint-Hyacinthe, Québec, J2S 2M2, Canada

## Abstract

The objective of this study was to quantify the reproductive performance of 4 reinsemination strategies in cows diagnosed nonpregnant using corpus luteum color flow Doppler ultrasonography on d 21 after last insemination. A total of 2,140 color flow Doppler ultrasonography exams from 845 Holstein cows from 10 commercial dairy herds were used in this study. Farms were visited every 2 wk by the research team. On d 7 after insemination, cows were enrolled in a randomized controlled trial to be assigned 1 of 4 treatments if they were subsequently diagnosed nonpregnant on d 21. All cows were then examined on d 21 using Doppler ultrasonography to diagnose nonpregnancy. Treatment A (CON) was the control group: nonpregnant cows were programmed to receive a standard Ovsynch protocol starting on d 32. Nonpregnant cows in treatment B (GnRH) were injected i.m. with GnRH on d 21 after insemination and reinseminated immediately. Cows in treatment C (2×GnRH) received an i.m. injection of GnRH on d 11 after insemination. If diagnosed nonpregnant on d 21 after insemination, they were injected i.m. with GnRH on d 21 after insemination and inseminated immediately. Cows in treatment D (Resynch) received an i.m. injection of GnRH on d 14 after insemination. If diagnosed nonpregnant on d 21 after insemination, they were injected i.m. with PGF_2α_ on d 21 after insemination and injected i.m. with GnRH on d 24. Then, a standard Ovsynch protocol was started on d 32. Statistical analyses were performed using mixed logistic regression models accounting for cow clustering and herd effect. A theoretical simulation was performed for each treatment to quantify the proportion of cows remaining nonpregnant 42 d after initial insemination. Of the 2,140 color flow Doppler ultrasonography exams in the study, 870 (40.6%) had a Doppler score of D0 (n = 444) or D1 (n = 426), which were indicative of nonpregnancy and used for data analysis. Overall, the number of exams assigned to each treatment was as follows: CON = 223 (25.6%), GnRH = 214 (24.6%), 2×GnRH = 220 (25.3%), and Resynch = 213 (24.5%). The final mixed multivariable logistic regression model included treatment, parity, days in milk at enrollment, and herd. Conception risk at first insemination following enrollment was 31.4, 20.6, 31.9, and 48.7% for treatments CON, GnRH, 2×GnRH, and Resynch, respectively. Based on the simulation, if 100 cows were diagnosed nonpregnant at their color flow Doppler ultrasonography exam on d 21 after last insemination, the total number of cows remaining nonpregnant 42 d after the initial insemination would be 69, 72, 58, and 51 for treatments CON, GnRH, 2×GnRH, and Resynch, respectively. Thus, the treatments used in groups 2×GnRH and Resynch for cows diagnosed nonpregnant on d 21 after insemination yielded better results than those used in the other groups.

## Highlights

• We compared 4 reinsemination strategies for use after a Doppler nonpregnancy diagnosis.
• Two of these strategies provided better results than the others.
• Benefiting from an early nonpregnancy diagnosis allowed improvements in subsequent reproduction.

## Graphical Abstract

**Figure:**
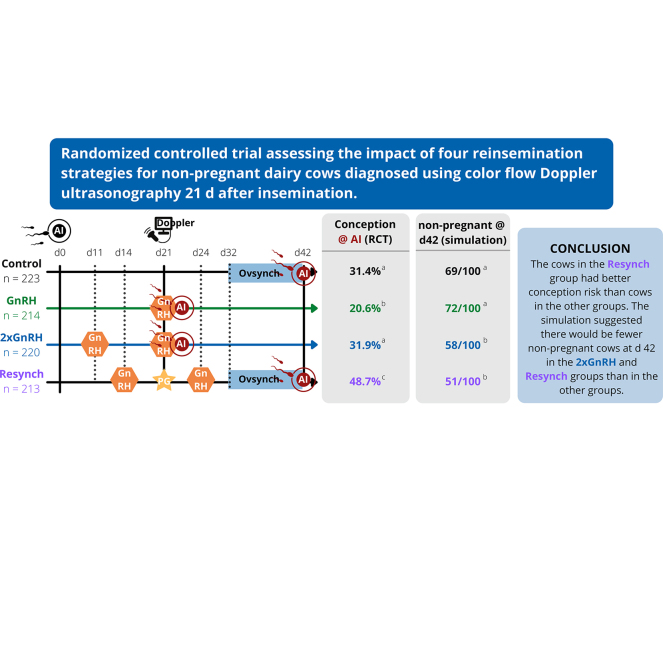
**Summary:** This study was conducted to compare 4 reinsemination strategies to be used when cows are diagnosed nonpregnant on d 21 after insemination using color flow Doppler ultrasonography. Statistical models and a theoretical simulation were used to quantify the effect on subsequent reproductive performance of cows. Although the 4 strategies did not have the same conception risk at first insemination following enrollment, some had shorter reinsemination delays than others. When applying these numbers to a theoretical simulation, 2 strategies (2×GnRH and Resynch) yielded a lower proportion of cows remaining nonpregnant 42 d after initial insemination.

An important aspect of reproductive management in dairy herds is to identify nonpregnant cows early to rebreed them (Fricke et al., 2016). As such, combining nonpregnancy diagnosis with strategies for reinseminating cows in a timely manner can improve reproductive efficiency and reduce days open (Fricke et al., 2016). Even if this can be applied intuitively at the cow level, it is important to keep in mind that applying it at the herd level remains the ultimate goal to optimize farm profitability.

The use of transrectal palpation of the uterus and B-mode ultrasonography is common on dairy farms to diagnose nonpregnancy in cows (Fricke et al., 2016). Color flow Doppler ultrasonography can also be used to quantify corpus luteum (**CL**) blood flow (Herzog et al., 2010; Bollwein et al., 2016) and identify nonpregnant cows on d 20 and 21 after last insemination (Siqueira et al., 2013; Dubuc et al., 2020). In such cases, the absence of blood flow in the CL reflects the nonpregnancy status.

For the use of color flow Doppler ultrasonography on dairy farms to become useful at the cow and herd levels, reinsemination strategies need to be developed and validated. Unfortunately, no data are currently available in the literature about this topic. To make them applicable widely at the farm level, these strategies would likely need to facilitate compliance (for instance, being always done on the same day of the week) and to optimize subsequent reproductive success.

Therefore, the objective of this study was to quantify the reproductive performance of 4 reinsemination strategies in cows diagnosed nonpregnant using CL color flow Doppler ultrasonography on d 21 after last insemination. The hypothesis was that some reinsemination strategies involving color flow Doppler ultrasonography yield better reproductive performance than those commonly used on dairy farms.

A randomized controlled trial was conducted between June 2016 and January 2019 in 10 commercial Holstein herds located in the vicinity of the Bovine Ambulatory Clinic of the Faculté de médecine vétérinaire of the Université de Montréal (St-Hyacinthe, QC, Canada). This research project was approved by the Animal Care Committee of the Université de Montréal (17-Rech-1878). The REFLECT statement was used to provide a standardized report of the results (O'Connor et al., 2010). Herd selection was based on convenience: for being located within a 1-h drive of the Bovine Ambulatory Clinic, for being willing to participate, for having an excellent history of compliance when using ovulation synchronization protocols, for using exclusively AI for insemination, and for using ovulation synchronization protocols for more than 90% of inseminations within the last 6 mo before the start of the study. The voluntary waiting period in these herds was 50 d.

Once herd owners agreed to participate in the study, farms were visited every 2 wk by an animal health technician and a veterinarian. Within a herd, farm visits were always performed on the same day of the week, and the day was chosen so that cows could be examined on d 7 and 21 after their last insemination. All cows meeting the latter criteria were systematically enrolled in the study. Cows could be enrolled more than once in the study but only once the 42-d period after initial insemination had passed. In such a case, it was considered a new enrollment. No nulliparous animals were enrolled. Thus, all cows on d 21 after last insemination were examined by transrectal ultrasonography (ExaGo, IMV Imaging) of the ovaries using the B-mode function (Rectal probe; frequency 7.5 MHz; dynamic range: 60 dB) by the same person. After identifying the presence of a CL (>10 mm in diameter), it was examined using color flow Doppler (frequency: 6.0 MHz; pulse repetition frequency: 4,000 Hz). Scoring of these CL was based on a semi-objective chart described elsewhere and shown to have an excellent accuracy to diagnose nonpregnancy in dairy cows (Dubuc et al., 2020). More specifically, CL were scored as D0, D1, D2, or D3 when 10% or less, between 11% and 30%, between 31% and 60%, or 61% or more of the surface was colored, respectively. Each CL was examined twice during the same transrectal palpation, and the highest Doppler score was used for this study. In cases where there were more than one CL in the same cow, the one with the highest Doppler score was used. Cows without any CL were not included in the present study.

In preparation for the next farm visit, cows on d 7 after the last insemination were randomly allocated to 1 of 4 treatments by the research team. Randomization was performed using a random number generator (Excel, Microsoft Corp.) and balanced within herds and within groups of 16 cows. Treatment allocation had to be done 2 wk before the color flow Doppler ultrasonography examination because some reinsemination strategies had intramuscular (i.m.) injections to be administered before the ultrasonography exam. Once cows were diagnosed nonpregnant with the color flow Doppler ultrasonography exams (scores D0 or D1) on d 21 after last insemination, cows followed the treatment allocation assigned 2 wk previously. Treatment A (**CON**) was the control group; these cows were programmed to receive a standard Ovsynch protocol starting on d 32 after last insemination, and cows were inseminated 10 d later on d 42 (Figure 1). Cows in treatment B (**GnRH**) were injected i.m. with GnRH (2 mL of Factrel, Zoetis) on d 21 after last insemination and inseminated immediately after (Figure 1). Cows in treatment C (**2×GnRH**) received an i.m. injection of GnRH (2 mL of Factrel, Zoetis) on d 11 after last insemination. If diagnosed nonpregnant on d 21 after last insemination, they were injected i.m. with GnRH (2 mL of Factrel, Zoetis) on d 21 after last insemination and inseminated immediately after (Figure 1). Cows in treatment D (**Resynch**) received an i.m. injection of GnRH (2 mL of Factrel, Zoetis) on d 14 after last insemination. If diagnosed nonpregnant on d 21 after last insemination, they were injected i.m. with PGF (5 mL of Lutalyse, Zoetis) on d 21 after last insemination and injected i.m. with GnRH (2 mL of Factrel, Zoetis) on d 24 after last insemination. Then, a standard Ovsynch protocol was started on d 32 after last insemination and cows were inseminated 10 d later on d 42 (Figure 1). Because of the requirement to perform injections, it was not possible to blind farmers to the treatment allocation.

**Figure 1:**
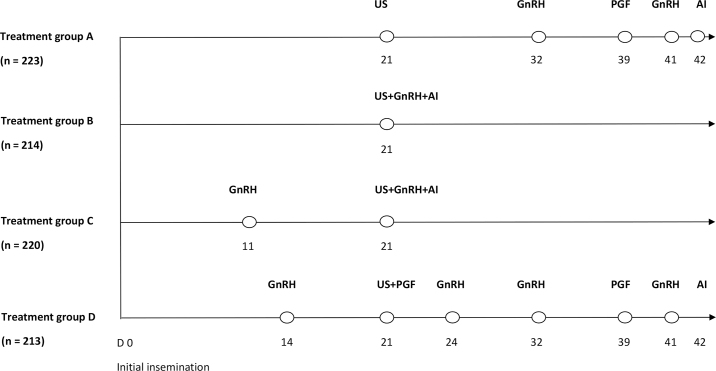
Reinsemination strategies for cows enrolled in a randomized controlled trial when diagnosed nonpregnant using the corpus luteum color flow Doppler ultrasonography (US) on d 21 after last insemination. Four treatments were allocated 2 wk before this examination. Based on the treatment assigned, cows received GnRH, PGF_2α_, and were inseminated (AI). Treatments: A (Control): nonpregnant cows received a standard Ovsynch protocol starting on d 32; B (GnRH): nonpregnant cows were injected i.m. with GnRH on d 21 after insemination and reinseminated immediately after; C (2×GnRH): cows received an i.m. injection of GnRH on d 11 after insemination. If diagnosed nonpregnant on d 21 after insemination, they were injected i.m. with GnRH on d 21 after insemination and inseminated immediately after; and D (Resynch): cows received an i.m. injection of GnRH on d 14 after insemination. If diagnosed nonpregnant on d 21 after insemination, they were injected i.m. with PGF_2α_ on d 21 after insemination and injected i.m. with GnRH on d 24.

All herds were visited every 2 wk by their regular herd veterinarian (blinded to study findings) to examine cows enrolled in treatment CON 32 d after insemination using transrectal palpation and B-mode ultrasonography. Cows were classified as pregnant (presence of embryonic vesicle with heartbeat) or nonpregnant. All nonpregnant cows from CON were systematically reinseminated using a standard Ovsynch protocol. Cows from the other groups were not examined at d 32. Reproductive and culling events of all cows were collected until 42 d after enrollment. Cows were eligible to be enrolled more than once in the study. Thus, the unit of interest in the study was every color flow Doppler ultrasonography exam.

A sample size of 178 color flow Doppler ultrasonography exams per treatment was estimated based on finding a difference of 15 percentage points (30% vs. 45%) in conception risk, with 95% confidence, 80% power, and 10% loss to follow-up (Dohoo et al., 2003). These numbers were based on a pilot study conducted to compare treatments CON and Resynch. Because we had 4 treatments and estimated that 33% of cows examined at d 21 after last insemination would have a score of D0 or D1, a total of 2,136 color flow Doppler ultrasonography exams was targeted for this study. The focus of the study was only nonpregnant cows.

Statistical analyses were performed using SAS software (version 9.3; SAS Institute Inc.). Descriptive statistics were calculated using Proc FREQ and Proc MEANS in SAS. Loss to follow-up and its reason were quantified. Univariable logistic regression models accounting for the clustering effect (random intercept) of cows (cows were eligible to be enrolled more than once) were built using Proc GLIMMIX in SAS; conception risk at first insemination following enrollment was the dependent variable of these models. Independent variables of interest were treatment (A, B, C, D), parity group (first, second, third or greater), DIM at enrollment, season of enrollment (summer: July–September, fall: October–December; winter: January–March; spring: April–June), and herd (1–10). All variables with *P* < 0.25 were retained for further modeling. A final multivariable mixed logistic regression model accounting for the clustering effect (random intercept) of cows was built using Proc GLIMMIX in SAS for conception risk at first insemination following enrollment (dependent variable) using a backward elimination strategy until only significant variables (*P* ≤ 0.05) were retained (herd was forced in the final model). The following variables were offered as fixed effect to the final model: treatment, season, parity group, DIM at enrollment, and herd. The retained variables in this mixed multivariable model were treatment, parity group, DIM at enrollment, and herd. Possible confounding variables were kept in the models if their effect was greater than 10% (Maldonado and Greenland, 1993). Least squares means were obtained from the final models, and differences between least squares means were computed using a Tukey-Kramer test.

To explore and provide practical information to potential users of these results, a theoretical simulation was then computed using an Excel spreadsheet and the least squares means (95% CI) obtained from the final model to estimate the proportion of cumulative conception 42 d after the initial insemination (35 d after enrollment) if a case where 100 cows were diagnosed nonpregnant at the color flow Doppler ultrasonography exam on d 21 (scores D0+D1). The simulation was built using conception risk at first insemination following enrollment and the theoretical possible number of inseminations during the 42-d period (treatments CON and Resynch had 1, treatments GnRH and 2×GnRH had 2). The first assumption used in this simulation was that 40.6% of cows being examined for the second time on d 42 (treatments GnRH and 2×GnRH had a second chance of being inseminated) were to be diagnosed nonpregnant using the color flow Doppler ultrasonography exam (scores D0+D1). This assumption was based on the proportion found in the present study and was the same for treatments GnRH and 2×GnRH. The second assumption was that all cows in the simulation would have a CL. The third assumption was that cows would only be inseminated according to the study treatment allocation (no heat detection performed). The fourth assumption was that cows in each treatment would have a conception risk identical to that reported in the present study. For treatments GnRH and 2×GnRH, we assumed that these cows would have been inseminated using the same treatment strategy (initially allocated) on d 42 after initial insemination; that is, as if it were applied systematically within a herd over time. The fifth assumption was that cows identified as D0 or D1 were really nonpregnant (assuming 100% sensitivity and specificity of the diagnostic test). In the end, this simulation provided several cows (out of 100) remaining nonpregnant 42 d after initial insemination. This approach was chosen to be able to compare fairly the 4 treatments over a 42-d period, even if they did not have the same number of possible inseminations. A Monte Carlo simulation (Gaussian distribution) in Excel was then used to calculate 500 random simulations of these numbers. Descriptive statistics (minimum, maximum, mean, standard deviation) from these simulations were computed. A Dunn's multiple comparison test was used to compare these means.

A total of 2,140 color flow Doppler ultrasonography exams from 845 Holstein cows from 10 commercial dairy herds were used in this study. The herd size ranged from 60 to 300 lactating cows (median = 100 cows). All herds were fed a TMR to meet their nutritional requirements. The 21-d pregnancy rate (based on a voluntary waiting period of 50 d) of these herds at the start of the study ranged from 15 to 28% (median = 22%). Half of the herds (n = 5) were housed in freestall barns, whereas the other half were housed in tiestall barns. No adverse events occurred during the study.

The median number of color flow Doppler ultrasonography exams per participating cow during the study was 3 (minimum: 1, first quartile: 2, third quartile: 6, maximum: 11). Of the 2,140 color flow Doppler ultrasonography exams in the study, 870 (40.6%) had a Doppler score of D0 (n = 444) or D1 (n = 426) and were used for data analysis. A total of 189 (8.8%) had 2 CL. Overall, the number of exams assigned to each treatment was as follows: CON = 223 (25.6%; from 211 cows), GnRH = 214 (24.6%; from 209 cows), 2×GnRH = 220 (25.3%; from 215 cows), and Resynch = 213 (24.5%; from 210 cows). When stratified by Doppler score, the numbers were CON = 114 (25.7%), GnRH = 108 (24.3%), 2×GnRH = 115 (25.9%), and Resynch = 107 (24.1%) for D0; and CON = 109 (25.6%), GnRH = 106 (24.9%), 2×GnRH = 105 (24.6%), and Resynch = 106 (24.9%) for D1. A total of 60 color flow Doppler ultrasonography exams had loss of follow-up, which were caused by culling (n = 38), do not breed status (n = 18), and mortality (n = 4). They represented 7.1% (n = 16), 7.0% (n = 15), 6.8% (n = 15), and 6.5% (n = 14) of loss to follow-up for treatments CON, GnRH, 2×GnRH, and Resynch, respectively. The median reinsemination delays (interval between initial insemination and subsequent insemination) for treatments CON, GnRH, 2×GnRH, and Resynch were 42, 21, 21, and 42 d, respectively.

The final multivariable mixed logistic regression model, where conception risk at the following insemination was the dependent variable, included treatment (*P* < 0.01), parity group (*P* < 0.01), DIM at enrollment (*P* = 0.04), and herd (*P* < 0.01). Least squares means from the final model of each treatment are presented in Figure 2.

**Figure 2:**
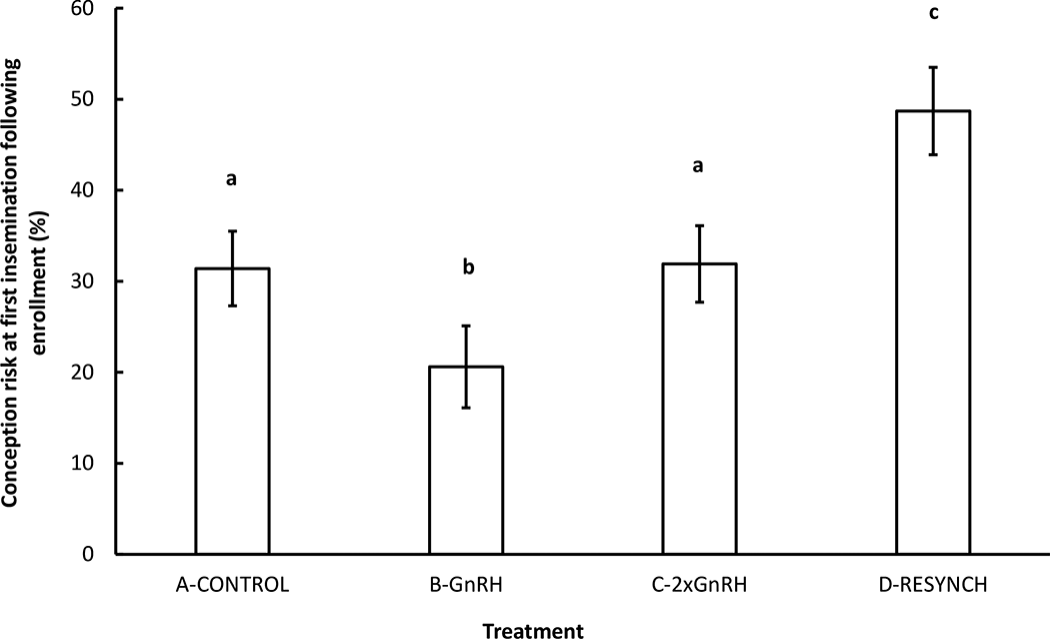
Least squares means (±SEM) of 4 reinsemination strategies from a randomized clinical trial conducted on 845 cows diagnosed nonpregnant using corpus luteum color flow Doppler ultrasonography on d 21 after last insemination in 10 herds (QC, Canada). Different letters (a–c) indicate a significant difference (*P* < 0.05). Treatments: A (Control): nonpregnant cows received a standard Ovsynch protocol starting on d 32; B (GnRH): nonpregnant cows were injected i.m. with GnRH on d 21 after insemination and reinseminated immediately after; C (2×GnRH): cows received an i.m. injection of GnRH on d 11 after insemination. If diagnosed nonpregnant on d 21 after insemination, they were injected i.m. with GnRH on d 21 after insemination and inseminated immediately after; and D (Resynch): cows received an i.m. injection of GnRH on d 14 after insemination. If diagnosed nonpregnant on d 21 after insemination, they were injected i.m. with PGF_2α_ on d 21 after insemination and injected i.m. with GnRH on d 24.

The theoretical simulation to quantify the proportion of nonpregnant cows 42 d after the initial insemination (35 d after enrollment) is presented in Figure 3. Based on this simulation, if 100 cows were diagnosed nonpregnant at their color flow Doppler ultrasonography exam on d 21 after last insemination, the total number of cows remaining nonpregnant 42 d after the initial insemination would be 69 (SD = 4, minimum = 57, maximum = 85), 72 (SD = 5, minimum = 55, maximum = 88), 58 (SD = 4, minimum = 45, maximum = 71), and 51 (SD = 5, minimum = 37, maximum = 67) for treatments CON, GnRH, 2×GnRH, and Resynch, respectively. These results show that treatments 2×GnRH and Resynch had a lower number of cows (*P* = 0.01) remaining nonpregnant 42 d after the initial insemination than the 2 other groups.

**Figure 3:**
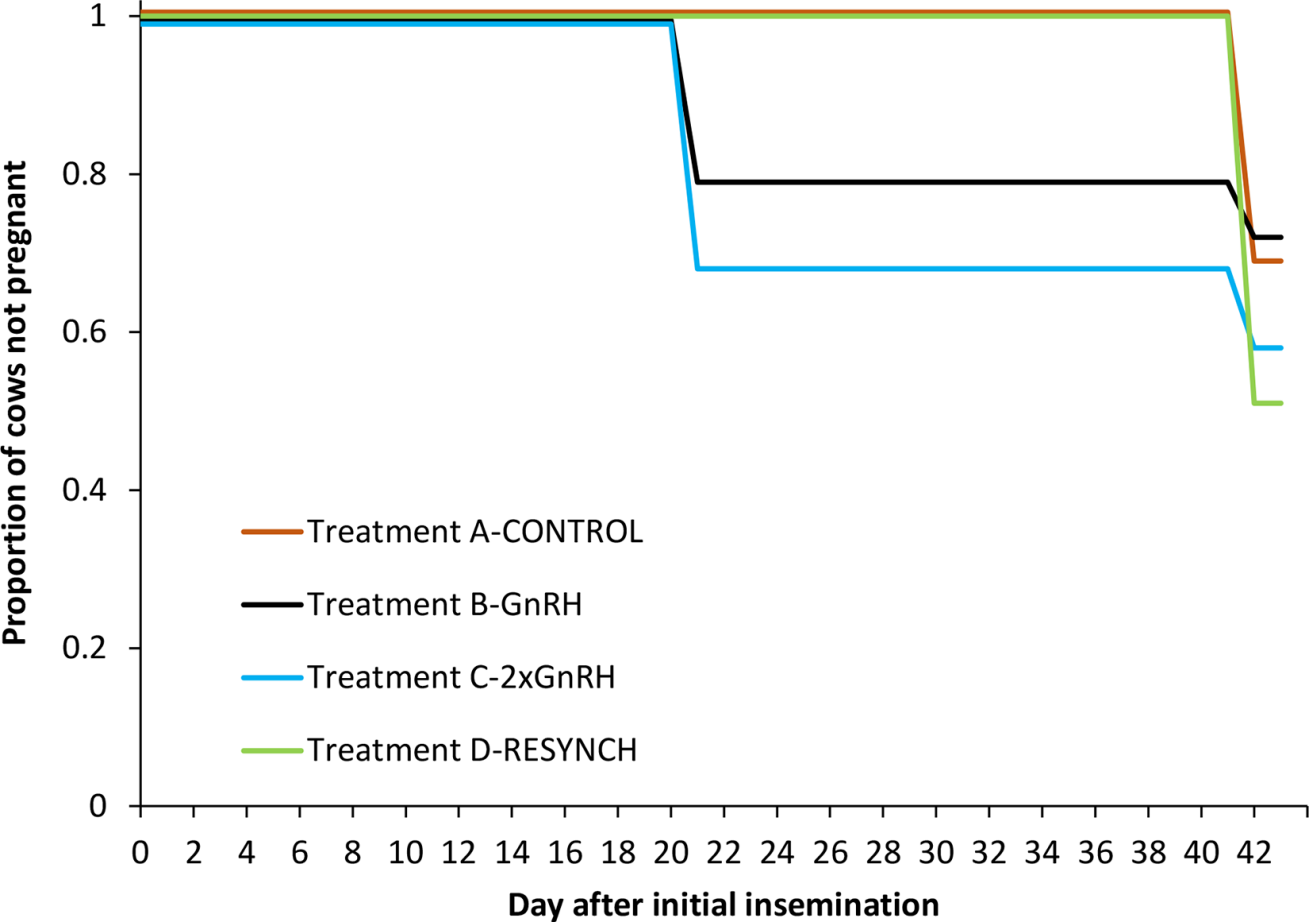
A theoretical simulation of the effect of 4 reinsemination strategies on the number of cows remaining nonpregnant 42 d after initial insemination for each of the treatments: CON, GnRH, 2×GnRH, and Resynch in cows diagnosed nonpregnant using corpus luteum color flow Doppler ultrasonography on d 21 after last insemination. Treatments: A (Control): nonpregnant cows received a standard Ovsynch protocol starting on d 32; B (GnRH): nonpregnant cows were injected i.m. with GnRH on d 21 after insemination and reinseminated immediately after; C (2×GnRH): cows received an i.m. injection of GnRH on d 11 after insemination. If diagnosed nonpregnant on d 21 after insemination, they were injected i.m. with GnRH on d 21 after insemination and inseminated immediately after; and D (Resynch): cows received an i.m. injection of GnRH on d 14 after insemination. If diagnosed nonpregnant on d 21 after insemination, they were injected i.m. with PGF_2α_ on d 21 after insemination and injected i.m. with GnRH on d 24.

Although CL color flow Doppler ultrasonography has been available for many years, few data are yet available about how to manage cows diagnosed nonpregnant using this diagnostic tool. Various research groups over time have been interested in finding a strategy (using B-mode ultrasonography or other tests) that leads to making a nonpregnancy diagnosis on d 21 after last insemination and rebreeding these cows (Green et al., 2010; Kelley et al., 2016). A lot of work has been done in the last 25 yr to optimize ovulation synchronization protocols in cows at their first insemination in a lactation (e.g., Ovsynch, Presynch, double-Ovsynch) and for reinseminating after a nonpregnancy diagnosis (Sani et al., 2011; Giordano et al., 2012; Martins et al., 2017). Of course, this body of literature inspired the arbitrary choice of our treatments. Because no data were available before the start of the present study, the chosen treatments were based on the hypothesis that they were likely to be easily implemented on commercial dairy farms. For instance, treatment CON was chosen because it is already commonly used on farms, and our results for this treatment were in line with what is reported elsewhere (Kelley et al., 2016). Treatment GnRH was designed to be as convenient as possible; that is, to reinseminate the cow on the same day as the nonpregnancy diagnosis on d 21. Not surprisingly, our results for this treatment were not good because it very likely led to poor synchrony of ovulation. The rationale behind including this treatment in the study was to provide an answer to farmers who really wanted to use that strategy. Treatment 2×GnRH was designed to be similar to a standard co-synch protocol with duration of 10 d and an assumed natural luteolysis occurring on d 18 after initial insemination (although we recognize that this might not hold true for many cows). Interestingly, conception risk of this treatment at first insemination after enrollment was similar to that of a standard Ovsynch protocol (Giordano et al., 2012). Because 2 inseminations are possible within a 42-d period when using this strategy, it deserves further investigation. Treatment Resynch was designed to be as similar as possible to a double-Ovsynch protocol, and our results are consistent with results from others when used for resynchronization (Giordano et al., 2012). One possible advantage of using Doppler ultrasonography would be to reduce the duration of the standard double-Ovsynch resynchronization protocol.

The answer to our study objective depends on what outcome is considered the most relevant. Our data showed that conception risk at first insemination following enrollment was higher in treatment Resynch than in all other groups. Although these findings are clinically relevant, we should not forget that GnRH and 2×GnRH treatments had median reinsemination delays 21 d shorter than those of CON and Resynch. Therefore, results from the theoretical and Monte Carlo simulations show that the number of cows remaining nonpregnant 42 d after initial insemination can vary. In fact, the 2×GnRH and Resynch treatments resulted in the fewest cows remaining nonpregnant 42 d after initial insemination. It is important to note that this simulation is imperfect compared with the real world, and it accounts for only a limited number of variables and assumptions. Thus, it should be interpreted with caution in accordance with these assumptions. The simulation helps capture the combined effect of conception risk and reinsemination delay, which is relatively close to a pregnancy rate over a 42-d period. Future studies exploring this aspect should consider making a comprehensive partial budget, as published elsewhere (Giordano et al., 2011), to better understand the financial impacts of implementing CL color flow Doppler ultrasonography.

Although the present study provides data on resynchronization of cows when using color flow Doppler ultrasonography on dairy farms for managing nonpregnant cows diagnosed on d 21 after last insemination, it has some weaknesses. First, selection of the participating herds was done by convenience and it remains unclear whether similar results would have been found if a larger or different herd population were selected. Clearly, inference of the study results is restricted to similar herds, including the fact that more than 90% of inseminations in these herds used ovulation synchronization protocols. In other words, these herds exclusively or almost exclusively used ovulation synchronization protocols for insemination. This aspect was crucial for the feasibility of the present study because it provided cohorts of cows inseminated on the same day every 14 d. In the context of dealing with relatively small dairy herds (which are very common in Québec and Eastern Canada), having sufficiently large groups of cows to examine during research farm visits was the only way to make this project economically and logistically feasible. Furthermore, this herd population would be the one targeted for implementing a Doppler reinsemination strategy (e.g., a sufficient number of animals to justify frequent farm visits, only using timed AI for insemination).

Another important point when interpreting the study results is that these herds did no or almost no heat detection (visual or with activity monitors), relying primarily on the use of timed AI to manage the reproduction of their herd. It remains unclear whether our results would be the same in a different reproduction management system. Future studies should explore this aspect as well as the herd-level effect of implementing systematic CL color flow Doppler ultrasonography exams on d 21 after last insemination on the herd 21-d pregnancy rate, as well as 21-d insemination rate and conception risk. This information would help users to quantify the potential benefit of implementing this strategy on dairy farms.

In conclusion, our results have shown that treatments 2×GnRH and Resynch used in cows diagnosed nonpregnant on d 21 after last insemination yielded better results than the control and GnRH treatments in herds using almost exclusively ovulation synchronization protocols for reproduction management. These data should be considered when implementing the use of CL color flow Doppler ultrasonography exams on farms to diagnose nonpregnancy in dairy cows.
